# Effects of Exergaming Tennis on Players’ Tennis Skills and Mental State Compared to Regular Tennis in Adult Players: Quasi-Experimental Study

**DOI:** 10.2196/73732

**Published:** 2026-02-02

**Authors:** Jia Sheng Ngo, See Ziau Hoe, Maziah Mat Rosly

**Affiliations:** 1 Department of Physiology Faculty of Medicine Universiti Malaya Kuala Lumpur Malaysia

**Keywords:** exergaming, tennis, sports skills, psychology, sport training

## Abstract

**Background:**

Exergaming, which combines physical activity with interactive gaming, has been shown to improve motor skills and fitness. However, exergaming’s potential in complex, open-skill sports such as tennis, which require real-time coordination, decision-making, and technical precision, remains underexplored. Furthermore, only a few studies have evaluated the impact of exergaming on both technical skill development and psychological outcomes such as motivation and confidence, especially among novice players. This study addresses these gaps by comparing the combination of exergame-based tennis training and on-court tennis training (EBTT+OCTT) with on-court tennis training alone (OCTT×2) in improving technical skills, grip strength, confidence, and motivation.

**Objective:**

This study aims to assess the effect of exergaming tennis in developing tennis technical skills, grip strength, confidence level, and motivation level.

**Methods:**

In a 12-week quasi-experimental trial, 66 novices were randomized to either the EBTT+OCTT or the OCTT×2 group. Participants consisted of 22 males and 44 females, with the mean BMI and age of approximately 22 (SD 2.9) kg/m^2^ and 26 (SD 7) years, respectively. The EBTT+OCTT group had 1 weekly session of OCTT and 1 session of exergaming training using Virtual Tennis (PlayStation 3 Move), while the OCTT×2 group completed 2 weekly on-court sessions. Outcomes included tennis technical skills such as groundstroke technique, service speed, and service accuracy (assessed using the Hewitt tennis test), grip strength (using a handheld dynamometer), confidence (using the Sports Confidence Inventory), and motivation (using the Sport Motivation Scale). Mean differences (MDs) between pre- and postintervention were collected and analyzed using repeated-measures ANOVA (α=.05) and Pearson correlation analysis. Effect sizes were calculated using partial eta squared (ηp²), where values ≥0.14 indicated large effects.

**Results:**

After 12 weeks of training, both EBTT+OCTT and OCTT×2 groups showed significant improvements (*P*<.001) in tennis technical skills (MD=17.06-22.62), grip strength (MD=9.59-11.04 kg), and confidence levels (MD=23.29-26.28). These outcome measures have large effect sizes (ηp²=0.84-0.92); however, they did not significantly differ (*P*>.05) across the groups when compared, with *P_grip strength_*=.24, *P_hit_*=.97, *P_accuracy_*=.86, *P_speed=_*.72, and *P_confidence_=*.31. In terms of motivation, EBTT+OCTT retained intrinsic motivation (IM) better than OCTT×2, with significant reductions in IM, mainly IM-to-know (MD=7, SD 2.95) and IM-to-accomplish (MD=5, SD 3.77) observed in the OCTT×2 group (*P*<.001). Grip strength, confidence, and motivation levels (except amotivation) showed positive correlations with tennis technical skills (*r*=0.39-0.80).

**Conclusions:**

EBTT+OCTT and OCTT×2 significantly improve tennis skills and confidence levels in novice players, although no significant differences were found between the two. However, EBTT+OCTT appeared to better sustain IM. Thus, EBTT+OCTT may serve as a supplementary tool for novice players to better learn tennis.

## Introduction

Technological innovations have facilitated the integration of physical activity through electronic platforms, particularly via exergaming—interactive video games that require physical movement for gameplay [1]. Substantial evidence demonstrates the efficacy of exergaming in enhancing motor coordination, reaction time, and physical fitness across various populations, including pediatric, geriatric, rehabilitation, and special needs cohorts [2,3]. These systems present novel opportunities for incorporating exercise into daily life, prompting investigation of their applications in sports training. According to the existing literature, most research has focused primarily on endurance and closed-skill sports (eg, cycling, running, and rowing), as their predictable, self-paced nature translates effectively to virtual environments [4-6]. In contrast, open-skill sports such as tennis have received limited attention due to the technical challenges of accurately replicating their complex biomechanics and decision-making demands in digital platforms [4]. Tennis demands precise mind-body coordination, decision-making skills, and real-time reactions [7]. While exergaming shows potential for early skills acquisition, the current literature lacks studies examining its effects in skill-based sports.

Tennis is a complex, open-skill sport that demands not only physical skill and execution of techniques such as serving and groundstrokes but also strong psychological attributes, including confidence and motivation [7,8]. Technical proficiency, such as accurate and powerful strokes, is fundamental to performance, yet these motor skills do not operate in isolation, and physical capability, such as grip strength, may also play a role in ensuring these skills are executed appropriately [4,9,10]. In addition, psychological readiness can influence how consistently and effectively players perform under pressure, make decisions, and maintain effort during training or competition [7,11].

While physical outcomes have been moderately investigated, there is a lack of research examining psychological outcomes, particularly confidence and motivation, which are known to influence motor learning and sports performance [4,7,11]. Confidence, for example, plays a key role in motor learning and performance; players with greater sport confidence are more likely to take risks, recover from mistakes, and execute techniques successfully under stress [7,12]. Similarly, motivation, especially intrinsic motivation (IM), affects the degree of engagement, persistence, and enjoyment during practice, which can directly enhance motor learning and skill retention [7,13]. These psychological factors are not only relevant to sport performance but may also be interrelated with technical skill development, making it important to study both domains simultaneously [7,11-13]. For instance, a motivated and confident player may be more willing to engage in deliberate practice, which leads to better technical improvement. Only a limited number of studies have concurrently examined the interrelationship between technical and psychological skills in an exergaming context, leaving a gap in understanding how these domains interact to support performance and engagement.

Exergaming, by blending physical movement with gaming elements, has the potential to impact both domains [1]. It can foster motor skill repetition in a stimulating environment while simultaneously supporting motivation and self-confidence through goal-setting and feedback [3]. However, most previous exergaming research has focused either on general fitness or cognitive outcomes, with limited exploration of its impact on sport-specific technical skills and psychological outcomes in a combined manner.

While both exergaming and traditional on-field exercises aim to promote physical fitness, they differ considerably in context, delivery, and user engagement. Research has consistently demonstrated that on-field exercises such as tennis, football, and badminton contribute to improved cardiovascular fitness, enhanced muscular strength, better body composition, and overall physical and mental well-being [8,14]. However, despite their benefits, on-field exercises can present certain challenges, such as time constraints, limited access to appropriate facilities, weather conditions, and personal motivation [14-16]. Exergaming, in contrast, combines physical movement with interactive digital gameplay, making exercise more accessible, enjoyable, and gamified [3]. Research has shown that adding exergaming to exercise regimes such as badminton, tennis, and golf can help encourage social interaction, allowing users to participate in cooperative or competitive activities with family, friends, or online communities, which may improve the appeal and sustainability of physical activity [17,18].

Tennis was chosen in this study because there is limited research examining this sport. There was also a need to evaluate whether skills learned through exergaming can transfer to real tennis performance. Although understanding the rules and strategies of tennis is important, mastery of the sport requires repeated physical practice to develop technical proficiency, coordination, and real-time decision-making skills. Exergaming may help players develop motor skills to respond to service and shots through repetitive practice, combining mental, visual, and motor learning.

Thus, the main objectives of this research were to determine whether integrating exergame-based tennis training and on-court tennis training (EBTT+OCTT) can help improve tennis technical skills, grip strength, confidence, and motivation levels compared with only the traditional on-court tennis training (OCTT×2). In addition, this research aimed to determine whether there is any correlation between grip strength, confidence, and motivation, to better understand their interrelationship and impact on tennis performance.

## Methods

### Study and Trial Designs

This quasi-experimental study was conducted in Malaysia, where participants were initially randomly allocated to either the EBTT+OCTT group or the OCTT×2 group as a parallel-group experimental study (aged 18 to 40 years, with balanced randomization in a ratio of 1:1). However, about 9%-10% (OCTT×2 [n=3] and EBTT+OCTT [n=4]) of participants in each group requested to change their assigned allocation, and these requests were accommodated to minimize participant dropout. These requests were made before commencement of the experimental procedure, and no participants were switched during the experimental period.

### Participants

Social media postings and word-of-mouth marketing were used to recruit research participants. Participants were predominantly urban residents, consisting of university students and young professionals from diverse socioeconomic strata. The sample size calculator program G*Power (version 3.1.9.4; Heinrich Heine University) was used to determine the sample size. A repeated measures ANOVA (within- and between-groups’ interactions) was selected as the statistical test. The calculation was based on a large effect size (f=0.4), a significance level of 0.05, and a power (1−β1) of 0.95 [19]. The large effect size was chosen based on previous literature in sports and exergaming interventions reporting medium to large effect sizes for motor and psychological outcomes [20,21]. In addition, as this study was exploratory and involved multiple dependent variables with expected training-related gains, a conservative large effect size was used to ensure adequate statistical power. Two groups (EBTT+OCTT and OCTT×2) and 2 measurement time points (pre- and posttraining) were included, with an estimated correlation of 0.5 between repeated measures. The analysis determined that a minimum of 24 participants (12 per group) were required. To account for a potential dropout rate of 20%, 29 participants were required in total. This study recruited 66 participants in total, with 33 participants assigned to each group (Textbox 1).

**The inclusion and exclusion criteria of the study.**

**Inclusion criteria**

- Individuals with less than 1 year of tennis experience.
- Aged between 18 and 40 years.
- No medical history or surgical history.
- Physical activity level below moderate as assessed by the International Physical Activity Questionnaire (IPAQ).

**Exclusion criteria**

- Individuals who are obese (BMI>30 kg/m^2^).
- Individuals who do not understand English or Malay.

In the recruitment process, 74 individuals were screened, and 66 met the inclusion criteria. The others were excluded for reasons such as having over a year of tennis experience (3 individuals), a high International Physical Activity Questionnaire (IPAQ) score (2 individuals), a BMI exceeding 30 kg/m² (2 individuals), or cardiorespiratory conditions such as asthma (1 individual). The final sample (22 males and 44 females with a mean age of 26.3 years) represented the university-affiliated and local recreational tennis community, which is roughly 60% female and predominantly aged 18 to 35 years. The participants’ weekly physical activity levels, averaging about 760 metabolic equivalents of task minutes on the IPAQ scale, aligned with those of a low-to-moderately active urban adult population. The low to moderate IPAQ score was selected because it can enhance the responsiveness to training interventions, as individuals with lower activity levels are more likely to demonstrate measurable improvements in physical and psychological outcomes [4,7]. Furthermore, exergaming interventions have been primarily designed for and shown to be particularly beneficial in low-active or novice populations, supporting the appropriateness of this inclusion criterion [7,22]. A standardized IPAQ score was also required to reduce variability in fitness and motor performance that might influence the responsiveness to training, particularly since the study targeted novice players [22].

### Experimental Procedure

Sixty-six individuals were divided into two groups, with a total of 33 individuals in each of the OCTT x 2 and EBTT+OCTT groups. The participants in the EBTT+OCTT group practiced tennis on both the exergaming platform and the traditional tennis court, whereas the OCTT×2 group practiced only on the traditional tennis court. Before the training started, the participants’ consent, demographic data, physical activity level via IPAQ, and baseline data such as sports motivation test, sports confidence inventory, grip strength via handheld dynamometer, and technical skills via the Hewitt tennis test were collected. All questionnaires and outcome measures were valid and reliable [22-26].

Both groups followed identical protocols for their on-court training sessions, which were conducted on tennis courts and with equipment meeting International Tennis Federation regulatory standards [27]. The sessions were done for 1.5 hours, comprising a warm-up period of 15 minutes, a 1-hour main practice, and a 15-minute cool-down. The exercise session duration and frequency were set following the American College of Sports Medicine guidelines [28,29]. The OCTT×2 group practiced on-court tennis twice a week, whereas the EBTT+OCTT group had one on-court and one exergaming session per week. In addition to the on-court tennis practice, participants in the EBTT+OCTT group also practiced tennis using a video game version of Virtual Tennis (Sports Champion 2, Zindagi Games, San Diego Studio) on a gaming device, the PlayStation 3 Move, for 1.5 hours.

**Figure 1:**
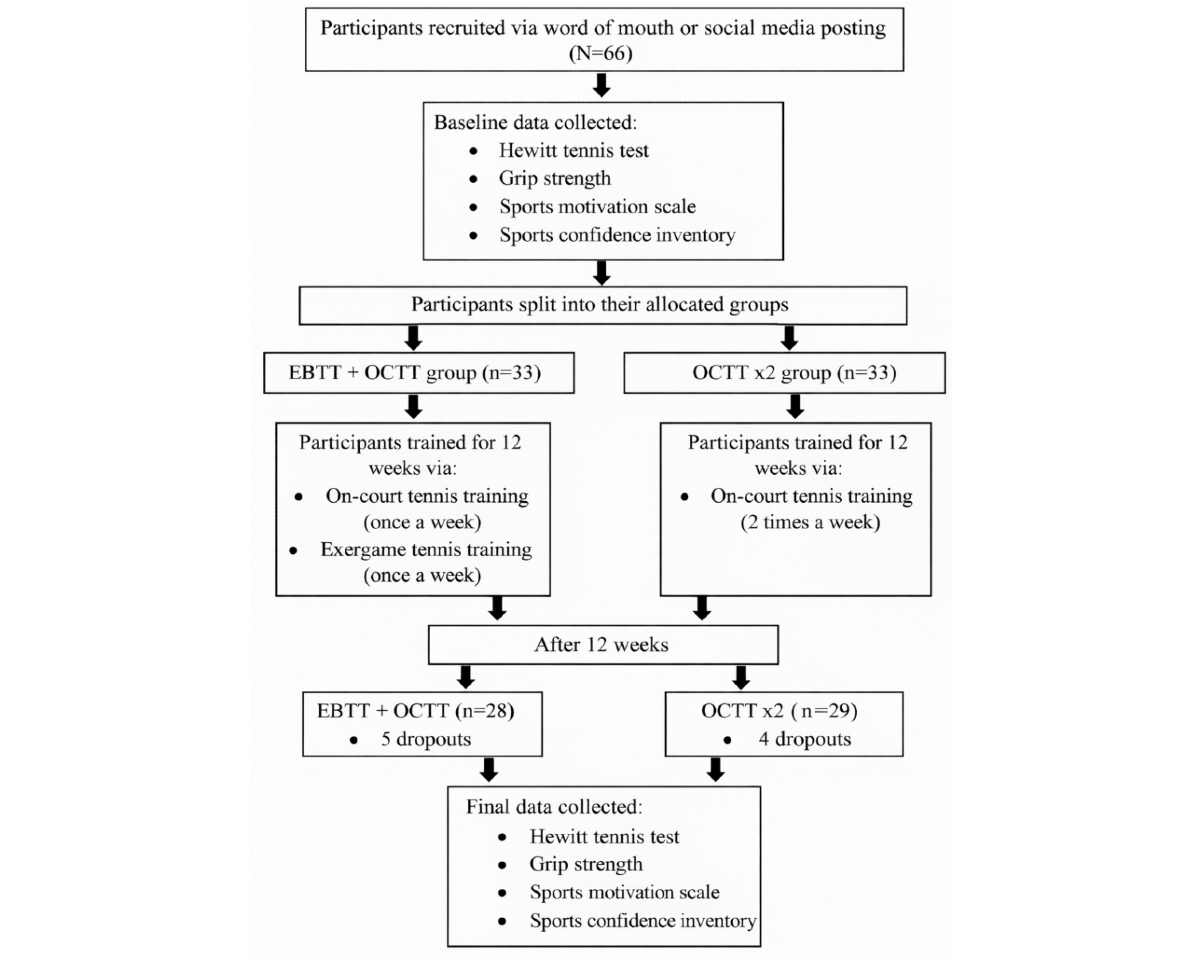
Flowchart of the quasi-experimental study. EBTT: exergame-based tennis training; OCTT: on-court tennis training.

Training included swing practices such as service, forehand, and backhand, and matches (either with a virtual avatar or among themselves). Matches were conducted between the 10th and 12th weeks. Both groups performed the task for 12 weeks (24 practice sessions). There was no group switching among participants during the experimental period. Four individuals in the OCTT×2 group and 5 in the EBTT+OCTT group dropped out during the second and third weeks, respectively, during the 12-week training period. Figure 1 provides the flowchart of the experimental design.

### Outcome Measures

#### Measurement of Tennis Technical Skills

In this study, the Hewitt tennis test was used to assess the player’s technical skills, such as service speed, service accuracy, and tennis strokes (forehand and backhand). This test was done pre- and post-12 weeks of practice. It has been demonstrated that this test is reliable and valid to conduct [25]. Participants in the tennis hit test aimed to score points by hitting balls into specific zones over the net, using forehand or backhand strokes during 10 trials. In the service accuracy and speed test, they served 10 balls into a target area, with accuracy scores based on placement and speed determined by the ball’s bounce distance after hitting the service court. Each subtest score is:

- Groundstroke (hit) score: participants earned 1-5 points per stroke based on where the ball landed on a court target zone. The maximum score for this test was 50.
- Service accuracy score: participants were instructed to serve into marked target zones on the court. Points were awarded based on the accuracy of placement, with a maximum of 50 points.
- Service speed score: speed was estimated based on the bounce distance of the ball in the service court, categorized into point zones, with a maximum possible score of 50.

All outcomes were treated as continuous variables (range: 0-50) and used in the statistical analysis. The normal value given by Hewitt’s Tennis test scores (score of 1-50) for beginners was graded from A (>18 points) to F (<3 points), where “A” represents an excellent score and “F” represents a very poor score [25]. While scoring the participant, the assessor was aware of the participant’s group allocation.

#### Measurement of Grip Strength

Measuring grip strength with handheld dynamometers was used due to their high validity and reliability [30,31]. Standardized protocols were followed to ensure accurate and comparable data. This typically involves a seated position with elbows flexed at 90 degrees, neutral wrists, and the handgrip aligned with the forearm [30,31]. Following the standardized protocol, 3 attempts were made for the dominant hand with rest intervals in between attempts. The highest value in kilograms (kg) was recorded and used for analysis. Grip strength was treated as a continuous variable in the statistical analysis. Measurements were taken pre- and post-12 weeks of training. The normal value for grip strength in Malaysia for males aged 18 to 54 ranged from 37 kg to 42 kg, whereas for females of a similar age, it ranged from 22 kg to 25 kg [32,33].

#### Measurement of Motivation and Confidence Level

The Sports Motivation Test (SMT) and the Sports Confidence Inventory (SCI) were used to measure motivation and confidence levels of participants when playing sports pre- and post-12 weeks of training. The SMT, developed by Pelletier et al [34], is a self-report questionnaire that assessed 7 types or subscales of motivation: IM-to-know, IM-to-accomplish, IM-to-experience stimulation, extrinsic motivation (EM)–identified, EM-introjected, EM–external regulation, and amotivation. Participants indicated the extent to which each statement reflected their motivation for engaging in sports, ranging from “not at all true” to “very true.” Each subscale score was calculated as the sum of all 4 items belonging to that subscale, resulting in 7 separate subscale scores ranging from 4 to 28. These subscale scores were used as separate continuous outcome variables in the analysis of motivation change over time and in correlation with tennis performance. The SMT is widely used in research settings to examine athletes’ motivation and understand the factors that drive their engagement in sports. High internal consistency is indicated by Cronbach alpha coefficients ranging from 0.70 to 0.90 [24,34].

The SCI, developed by Vealey [35], is a widely used questionnaire designed to assess athletes’ self-confidence in sports situations. Participants rate their level of confidence in each statement on a scale ranging from “strongly disagree” to “strongly agree.” The SCI measures both general and sport-specific self-confidence across various domains, such as skill execution, physical conditioning, and resiliency [23]. The total confidence score ranges from 8 to 72, with higher scores indicating greater sport-specific confidence. All 8 items were summed to calculate a total confidence score, which was used in pre-post comparisons and correlation analyses as a continuous variable. According to reliability assessments, the SCI has a high degree of internal consistency, with Cronbach alpha values between 0.70 and 0.90 [35]. Test-retest reliability has also been established, demonstrating stability over time.

### Data Analysis

SPSS software, created by International Business Machines, was used to gather and analyze the data. Normality was evaluated using the Shapiro-Wilk test, which is highly effective at detecting nonnormality [36]. For normally distributed data, repeated measures ANOVA was performed. Final analysis was conducted per protocol outlined in the “Data Analysis” section, where participants were analyzed according to the group they actually participated in, instead of their original randomized groups. In addition, a modified intention-to-treat (ITT) analysis was also performed, in which all participants who were randomized initially and completed the study were included in the analysis according to their originally assigned groups. This analytical approach excludes missing data from participants who dropped out of their respective interventions. To evaluate whether the treatment effects differed significantly between modified ITT analyses and per-protocol analyses, treatment effects from both approaches were compared. The primary comparison was based on the magnitude and 95% CIs of the treatment effects. Consistency between modified ITT and per-protocol analyses effects was interpreted as evidence of robustness. Effect sizes were evaluated using partial eta squared (ηp²), with results classified as small (0.01-0.06), medium (0.06-0.14), or large (≥0.14) [37]. Partial eta squared (ηp²) was used as the measure of effect size for the repeated-measures ANOVA results, as it is appropriate for estimating effect sizes in factorial and within-subject designs. Unlike Cohen *d*, which is suited for simple 2-group comparisons, ηp² provides a proportion of variance explained by each effect (main and interaction), making it more suitable for analyzing group × time interactions in repeated-measures designs [37]. If the time (pre-post training) and group interaction are significant, posthoc pairwise comparisons were used; however, if only the time effect is significant, but there is no interaction between time and group, paired *t* tests were used to confirm improvement or mean difference (MD) in each group. A 95% CI of the MD was also calculated to determine the range within which the true difference between population means is likely to fall. The correlation between the changes (differences between pre- and postintervention) in players’ grip strength, confidence, and motivation levels with their tennis technical skills was analyzed using Pearson’s product-moment correlation coefficient (*r*). According to Cohen [38], the strength of the relationship is weak (*r*=0.10), moderate (*r*=0.30), or strong (r=0.50). Pearson *r* value was squared and multiplied by 100 (*r*^2^) to determine the percentage of variation explained by 1 variable for another. In this study, *r*² was used to determine the percentage of variability in tennis technical skills accounted for by grip strength, confidence, and motivation levels. Findings with a *P*<.05 were considered statistically significant.

### Ethical Considerations

This study involving human participants was reviewed and approved by the Universiti Malaya Research Ethics Committee, Malaysia. The ethics approval was granted on January 26, 2023 (UM.TNC2/UMREC_2286). Written informed consent was obtained from all participants before enrollment in the study. Participants were informed about the study objectives, procedures, potential risks and benefits, and their right to withdraw at any time without penalty. The study did not involve any secondary use of existing data; all data were collected prospectively as part of this trial. To ensure participant privacy and data confidentiality, all data collected remained anonymous by assigning unique participant codes. Personal identifiers were not linked to the study data. Electronic records were stored securely on password-protected devices. Participants did not receive any monetary compensation for their participation. However, they were provided with gift vouchers from local grocery stores and restaurants and were allowed to retain access to the tennis training facilities during the study period at no cost, as a token of appreciation for their involvement. Written informed consents were also obtained from participants when their images were used in the papers. This trial was not preregistered, as it did not measure any clinical outcomes.

## Results

### Overview

The results are provided in 2 parts: the first part is the comparative analysis of the physical variables (tennis technical skills and grip strength) and psychological variables (confidence and motivation levels) between EBTT+OCTT and OCTT×2, and the second part is the correlation analysis of the relationships between grip strength, confidence, and motivation with the tennis technical skills. All variables are presented as mean and SD for parametric data. Demographic data, including BMI, age, sex, and physical activity level, were collected. Independent *t* tests revealed no significant differences between groups in age, training experience, sex (male and female), BMI, and physical activity levels (Table 1).

**Table 1:** Summary of demographic data for the quasi-experimental study.

Parameter			EBTT+OCTT^a^ (n=33)		OCTT×2^b^ (n=33)		*P* value
Age (y), mean (SD)			26.82 (7.08)		25.85 (6.15)		.64
**Sex, n (%)**							
	Male	10 (30)		12 (36)		.68	
	Female	23 (70)		21 (64)		.79	
Training experience (mo), mean (SD)			3.3 (2.44)		2.9 (3.12)		.86
Height (m), mean (SD)			1.64 (0.66)		1.65 (0.58)		.34
Weight (kg), mean (SD)			60.1 (7.8)		59.6 (7.7)		.54
BMI (kg/m^2^), mean (SD)			22.35 (2.94)		21.89 (2.90)		.57
International Physical Activity Questionnaire (METs^c^), mean (SD)			808.94 (300.97)		712.35 (311.81)		.43
Dropouts, n (%)			5 (15)		4 (12)		—^d^

^a^EBTT+OCTT: exergame-based tennis training with on-court tennis training.

^b^OCTT: on-court tennis training.

^c^MET: metabolic equivalent of task.

^d^Not applicable.

Data collections were conducted from January to April 2024. During the 2nd and 3rd weeks of the study, 4 participants from the OCTT×2 group and 5 from the EBTT+OCTT group dropped out due to work schedules, health issues, transportation difficulties, or family emergencies (Table 1). The data of participants who dropped out were not used in the analysis. Shapiro-Wilk normality tests indicated that the differences between paired variables in the EBTT+OCTT and OCTT×2 groups were not statistically significant (*P*>.05), confirming that all paired variables were normally distributed.

In general, the treatment effect estimates from the modified ITT and per-protocol analyses were generally consistent for all outcome measures. The outcome measures showed overlapping 95% CIs and similar significance levels (P) between the 2 analyses, indicating no meaningful difference between the 2 analytical approaches. The table on the modified ITT analysis is provided in Multimedia Appendix 1.

### Effects of EBTT+OCTT and OCTT×2 on Physical and Psychological Variables

#### Effect of EBTT+OCTT and OCTT×2 on Grip Strength

Both groups showed a significant improvement (*P*<.001) with a large effect size (ηp²=0.84) from pre- to posttraining (Table 2). A total of 28 of 33 participants in the EBTT+OCTT group have an increase in grip strength by 1.5 times or 11.04 kg on average, while 29 of 33 participants in the OCTT×2 group improved their grip strength by 1.6 times or an average of 9.59 kg. However, grip strength showed no significant difference between groups (*P*=.24) with a small effect size (ηp^2^=0.03).

**Table 2:** Comparison of grip strength, hit scores, accuracy scores, speed scores, and confidence level within and between groups.

Outcome measure and group		Pretest, mean (SD)	Posttest, mean (SD)	Value, MD^a^ (95% CI; % changed)	Within-group *P* value		Between groups *P* value	
**Grip strength**						<.001		.24
	EBTT+OCTT^b^	21.68 (14.96)	32.71 (0.82)	11.04 (9.24-12.83; 51.10)				
	OCTT^c^×2	16.10 (13.97)	25.69 (0.68)	9.59 (7.86-11.31; 59.57)				
**Hit scores**						<.001		.97
	EBTT+OCTT	4.32 (4.68)	21.46 (7.21)	17.14 (14.95-19.34; 400)				
	OCTT×2	5.31 (5.41)	22.38 (9.23)	17.06 (14.27-19.86; 321.28)				
**Accuracy scores**						<.001		.86
	EBTT+OCTT	4.21 (3.25)	26.54 (8.33)	22.32 (19.55-25.09; 530.17)				
	OCTT×2	4.66 (3.30)	27.28 (7.51)	22.62 (20.38-24.86; 485.41)				
**Speed scores**						<.001		.72
	EBTT+OCTT	3.50 (2.92)	21.25 (6.03)	17.75 (15.62-19.88; 507.14)				
	OCTT×2	2.97 (2.16)	20.17 (6.34)	17.21 (14.97-19.44; 579.46)				
**Confidence level**						<.001		.31
	EBTT+OCTT	37.04 (9.21)	60.32 (2.90)	23.29 (18.93-27.64; 62.88)				
	OCTT×2	34.17 (7.31)	60.45 (3.80)	26.28 (22.19-30.36; 76.91)				

^a^MD: mean difference.

^b^EBTT+OCTT: exergame-based tennis training with on-court tennis training.

^c^OCTT: on-court tennis training.

#### Effect of EBTT+OCTT and OCTT×2 on Technical Skills

Technical skills were categorized into 3 measures: forehand and backhand (hit) score, service accuracy, and service speed. Significant differences (*P*<.001) were found in all 3 measures, pre- and posttraining, for both groups, with large effect sizes: ηp²_hit_=0.88, ηp²_accuracy_=0.92, and ηp²_speed_=0.91. In the EBTT+OCTT group, 28 of 33 participants’ hit score increased by 5 times (MD 17.14), service accuracy by 6.3 times (MD 22.32), and service speed by 6.1 times (MD 17.75). In the OCTT×2 group, 29 of 33 participants’ hit score increased by 4.2 times (MD 17.06), service accuracy by 5.9 times (MD 22.62), and service speed by 6.8 times (MD 17.21). However, for all technical skill outcome measures, there were no significant differences between groups (*P*_hit_=.97, *P*_accuracy_=.86, and *P*_speed_=.72), with small effect sizes (ηp²_hit_=0.01, ηp²_accuracy_=0.03, and ηp²_speed_=0.01) (Table 2).

#### Effect of EBTT+OCTT and OCTT×2 on Confidence Level

The main effect of time was significant (*P*<.001) with a large effect size (ηp²=0.84), indicating that participants in both groups showed significant improvement from pre- to posttraining. In the EBTT+OCTT group, 28 of 33 participants improved their confidence level by 23.29 points (~1.6 times on average), whereas in the OCTT×2 group, 29 of 33 participants increased by 26.28 points (~1.8 times) compared with baseline. However, the interaction between pre-post training and the group was not significant (*P*=.31) with a small effect size of 0.02, suggesting no overall difference between the EBTT+OCTT and OCTT×2 groups (Table 2).

#### Effect of EBTT+OCTT and OCTT×2 on Motivation Level

Overall, 28 of 33 participants in the EBTT+OCTT group showed a significant decrease in IM-to-accomplish, EM-identified, and EM-introjected. Whereas 29 of 33 participants in the OCTT×2 group, other than a significant increase in amotivation level, there were significant decreases in IM-to-know, IM-to-accomplish, EM-identified, EM-introjected, and EM–external regulation. In terms of the comparison between groups, the reduction of IM-to-know score and IM-to-accomplish score was greater in the OCTT×2 group than the EBTT+OCTT group, leading to a statistical significance, *P*<.001, when compared. A large effect size was also found, ηp² (IM-to-know)=0.25, and ηp² (IM-to-accomplish)=0.12. The only finding in EM that was statistically significant (*P*<.001) with a large effect size of ηp²=0.30 was the decrease in the EM–external regulation score of OCTT×2 in comparison to EBTT+OCTT (Table 3).

### Relationship Between Grip Strength, Confidence, and Motivation With Tennis Technical Skills

#### Relationship Between Grip Strength and Tennis Technical Skill

The bivariate correlation between grip strength and tennis hit score was significantly positive (r=0.70; *P*<.001). Grip strength was also significantly positively correlated with service accuracy (r=0.45; *P*<.001) and service speed (r=0.42; *P*=.001). The proportion of variance (r²) explained by grip strength for tennis hit score, service accuracy, and service speed was 0.50, 0.20, and 0.17, respectively.

**Table 3:** Comparison of different motivation levels within and between groups.

Outcome measures			Pretest, mean (SD)		Posttest, mean (SD)		Value, MD^a^ (95% CI; % changed)		Within-group *P* value		Between groups *P* value	
**IM^b^-to-know**												<.001
	EBTT+OCTT^c^	24.18 (2.92)		22.39 (4.12)		–1.79 (–3.85 to 0.28; 7.40)		.04				
	OCTT^d^×2	24.21 (2.53)		17.21 (2.88)		–7 (–8.43 to 5.57; 28.91)		<.001				
**IM-to-accomplish**												<.001
	EBTT+OCTT	23.29 (2.57)		21.82 (2.21)		–1.46 (–2.90 to –0.03; 6.27)		.04				
	OCTT×2	24.38 (2.21)		18.41 (2.21)		–5.97 (–7.09 to 4.85; 24.49)		<.001				
**IM-to-experience stimulation**												.66
	EBTT+OCTT	24.50 (2.66)		24.18 (2.71)		–0.32 (–1.78 to 1.14; 1.31)		.66				
	OCTT×2	24.21 (2.37)		24.31 (2.71)		0.10 (–1.26 to 1.47; 0.41)		.88				
**EM^e^-identified**												.42
	EBTT+OCTT	24.54 (2.62)		19.11 (2.50)		–5.43 (–6.72 to –4.13; 22.13)		<.001				
	OCTT×2	24.14 (2.84)		19.41 (2.69)		–4.72 (–5.95 to –3.50; 19.55)		<.001				
**EM-introjected**												.93
	EBTT+OCTT	17.25 (3.40)		14.64 (2.83)		–2.61 (–4.35 to –0.86; 15.13)		.01				
	OCTT×2	17.17 (4.48)		14.69 (3.39)		–2.48 (–4.53 to –0.43; 14.44)		.02				
**EM–external regulation**												<.001
	EBTT+OCTT	24.54 (2.83)		23.11 (2.60)		–1.43 (–2.99 to 0.13; 5.83)		.07				
	OCTT×2	24.34 (2.79)		18.24 (2.40)		–6.10 (–7.29 to –4.92; 25.06)		<.001				
**Amotivation**												.91
	EBTT+OCTT	13.68 (2.98)		20.76 (2.06)		7.08 (6.31 to 9.04; 51.75)		.06				
	OCTT×2	13.86 (3.16)		21.66 (2.44)		7.79 (6.20 to 9.38; 56.20)		<.001				

^a^MD: mean difference.

^b^IM: intrinsic motivation.

^c^EBTT+OCTT: exergame-based tennis training with on-court tennis training.

^d^OCTT: on-court tennis training.

^e^EM: extrinsic motivation.

#### Relationship Between Confidence Level and Tennis Technical Skill

For confidence, the bivariate correlation with tennis hit score (*r*=0.70; *P*<.001) and service speed (*r*=0.33; *P*=.01) was positive and significant. However, the correlation between confidence and service accuracy was not significant (*r*=0.21; *P*=.12). The coefficient of determination (*r*²) for confidence with tennis hit score and service speed were 0.50 and 0.11, respectively.

#### Relationship Between Motivation Level and Tennis Technical Skill

IM had significant positive correlations with tennis hit score and service accuracy. The correlations with IM-to-know, IM-to-accomplish, and IM-to-experience stimulation were *r*=0.60, 0.45, and 0.80 for tennis hit score, and *r*=0.35, 0.40, and 0.40 for service accuracy. However, there was no significant correlation between tennis service speed score and IM-to-know, IM-to-accomplish, and IM-to-experience, where *r*=0.21 (*P=*.11), 0.18 (*P=.*18), and 0.18 (*P=*.18), respectively. The *r*² values for tennis hit score and IM-to-know, IM-to-accomplish, and IM-to-experience stimulation were 0.36, 0.21, and 0.62, respectively. For service accuracy, the *r*² values were 0.12, 0.16, and 0.16.

EM showed significant correlations (*P*<.001) only with tennis hit score, with r = 0.60 (EM-identified), *r*=0.67 (EM-introjected), and *r*=0.64 (EM–external regulation). There were no significant correlations with service speed (*P*_EM-identified_=.21, *P*_EM-introjected_=.72, and *P*_EM–external regulation_=.65) or accuracy (*P*_EM-identified_=.86, *P*_EM-introjected_=.26, and *P*_EM–external regulation_=.45). The *r*² values for tennis hit score and EM-identified, EM-introjected, and EM–external regulation were 0.36, 0.45, and 0.41, respectively.

Finally, amotivation was negatively correlated with tennis hit score (*r*=–0.39; *P*<.001), but there were no significant correlations with service speed (*P*=.46) or accuracy (*P*=.60). The *r*² between tennis hit score and amotivation was 0.15, meaning that 15% of the variability in tennis hit scores could be predicted by amotivation. Table 4 provides the correlation between grip strength, confidence, and motivation levels with tennis technical skills.

**Table 4:** Correlation analysis (Pearson r and 2-tailed *P* value) between grip strength, confidence, and motivation levels with tennis technical skills.

Outcome measures			Hit scores		Accuracy scores		Speed scores
**Grip strength**							
	*r*	0.70^a^		0.45^a^		0.42^a^	
	*P* value	<.001		<.001		.001	
**Confidence level**							
	*r*	0.70^a^		0.21		0.33^a^	
	*P* value	<.001		.12		.01	
**IM^b^-to-know**							
	*r*	0.60^a^		0.35^a^		0.21	
	*P* value	<.001		.01		.11	
**IM-to-accomplish**							
	*r*	0.45^a^		0.40^a^		0.18	
	*P* value	<.001		<.001		.18	
**IM-to-experience stimulation**							
	*r*	0.80^a^		0.40^a^		0.18	
	*P* value	<.001		<.001		.18	
**EM^c^-identified**							
	*r*	0.60^a^		0.17		–0.02	
	*P* value	<.001		.21		.86	
**EM-introjected**							
	*r*	0.67^a^		0.05		–0.15	
	*P* value	<.001		.72		.26	
**EM–external regulation**							
	*r*	0.64^a^		0.06		–0.10	
	*P* value	<.001		.65		.45	
**Amotivation**							
	*r*	–0.39^a^		–0.07		–0.10	
	*P* value	<.001		.60		.46	

^a^The correlation is significant at a significant level of .05 (2-tailed).

^b^IM: intrinsic motivation.

^c^EM: extrinsic motivation.

## Discussion

### Principal Findings

In general, both EBTT+OCTT and OCTT×2 groups demonstrated significant improvements (*P*<.001) in tennis technical skills, all with large effect sizes, though no significant between-group differences were found across hit scores (*P*=.97), service accuracy (*P*=.86), and service speed (*P*=.72). The absence of significant between-group differences should not be interpreted as evidence of equivalence between the interventions, as this study was not designed or powered to assess equivalence or noninferiority. Grip strength increased significantly (*P*<.001) in both groups, with no significant interaction effects (*P*=.24). Confidence levels also improved significantly (*P*<.001) in both groups, but without significant between-group differences (*P*=.31). In terms of motivation, the EBTT+OCTT group showed significant decreases (*P*<.001) in EM-identified and EM-introjected, while the OCTT×2 group showed significant reductions (*P*<.001) in IM-to-know, IM-to-accomplish, EM-identified, and EM–external regulation. Notably, the OCTT×2 group experienced significantly greater decreases in IM-to-know, IM-to-accomplish, and EM–external regulation compared to EBTT+OCTT, all with large effect sizes. Correlational analysis revealed strong positive relationships (*r*>0.50) between hit skill and grip strength, confidence level, IM-to-know, IM-to-experience stimulation, and all EM subscales. However, a negative correlation was observed between hit skill and amotivation (*r*=–0.39).

### Comparison With Previous Work

#### Effect of Exergaming on Grip Strength

This study found significant improvements in grip strength in both EBTT+OCTT and OCTT×2 groups. Grip strength increased by 1.5 times in the EBTT+OCTT group and by 1.6 times in the OCTT×2 group. These results align with previous research on sports training and muscular strength. The likely mechanism is the repeated high-intensity activation of the forearm and hand muscles required for racket control and stroke execution. A previous study and a review have corroborated that regular engagement in sports requiring sustained gripping actions and upper limb coordination, such as tennis, induces significant neuromuscular adaptations that manifest as measurable strength gains in distal upper extremity musculature [9,39]. The observed grip strength improvements in the EBTT+OCTT group (1.5 times) demonstrated comparability with findings from an electromyography biofeedback exergaming study, which reported a 0.17 times enhancement in grip strength following training [40]. The substantially greater improvements observed in this quasi-experimental study may be attributable to the longer intervention duration (12 weeks vs 2 weeks), allowing for more pronounced neuromuscular adaptation. These results support the theoretical framework suggesting that exergaming systems designed to replicate sport-specific movements can elicit muscular engagement patterns and strength development comparable to conventional training modalities [40]. While the between-group comparison revealed no statistically significant difference in grip strength improvement, the marginally lower gain in the EBTT+OCTT condition may reflect biomechanical differences in equipment characteristics. Specifically, the substantially lighter mass of the exergaming controller (90 g) compared to a standard tennis racket (280 g-300 g) potentially reduced the mechanical loading and strength demands during gameplay. Nevertheless, the significant improvements observed in the EBTT+OCTT group suggest that exergaming tennis, while not specifically designed for grip strengthening, can effectively enhance grip strength through repeated sport-specific movement patterns. These findings expand current understanding of EBTT+OCTT’s therapeutic potential and its capacity to produce physiological adaptations similar to traditional training approaches.

#### Effect of Exergaming on Technical Skills

This study showed significant improvements in technical skill scores, with large effect sizes in both EBTT+OCTT and OCTT×2 groups. These results suggest that exergaming and traditional training are both effective for enhancing tennis skills. Improvements were likely driven by repetitive practice, whether in virtual or real settings, which aligns with previous research on motor learning in neurological rehabilitation [41]. This reflects the motor learning principle of practice, where skills must be rehearsed correctly to be mastered [42].

In the EBTT+OCTT group, hit scores increased by 5.5 times, service accuracy by 6.3 times, and service speed by 6.1 times. In the OCTT×2 group, hit scores rose by 4.2 times, service accuracy by 5.9 times, and service speed by 6.8 times. These findings are consistent with earlier studies, showing that exergaming can enhance motor skills and physical performance comparably to on-court training [42-45]. For example, one study reported that both exergaming and on-court training improved tennis players’ reaction time by 1.03 times, indicating that exergaming may help players prepare for incoming shots [43]. Similar results have been observed in children, where exergaming improved forehand and backhand skills by 1.5 times compared to 1.33 times in traditional training [44]. The greater improvement observed in this study (average skill increase of 5.5 times) compared with previous work (around 1.3 times) may be explained by the novice status of participants. Before the training, their baseline skills were poor, scoring below 6 of 50 on the Hewitt tennis test. However, with structured guidance and consistent practice, their skills improved dramatically over 12 weeks. This reflects the principle of specificity, where training effects are specific to the practiced activity [46]. As EBTT+OCTT replicates tennis movements, skill gains in the virtual setting were transferred to real-world performance.

Supporting evidence comes from studies in other domains. Exergaming-based balance training improved balance by 1.67 times, similar to conventional balance training [47]. Another study on adolescent tennis players reported a 0.38-times increase in Universal Tennis Rating when training combined with virtual reality and tablet-based cognitive drills was compared to regular training [45]. Collectively, these findings highlight that exergaming can be as effective as traditional training for enhancing technical performance. The shared success likely stems from common motor learning principles and practice specificity [46,47]. Finally, this study extends the literature by focusing on novice players. Exergaming provided an engaging, space-efficient alternative for skill development, requiring only about 4×3 meters of training space.

#### Effect of Exergaming on Confidence Level

The study found that both EBTT+OCTT and OCTT×2 significantly improved participants’ confidence in tennis performance. Confidence increased by 1.6 times in the EBTT+OCTT group and by 1.8 times in the OCTT×2 group. These results align with the self-efficacy theory, which highlights mastery experiences as a core source of confidence [48]. In this trial, structured and repetitive practice enabled participants to progressively master tennis skills, enhancing their belief in their abilities. The large effect sizes further indicate that both training types were highly effective in fostering a sense of control over performance, consistent with self-efficacy principles [49].

Previous research supports this view, showing that repeated practice in real sports settings increased athletes’ confidence by 1.21 times, thus preparing them for competition [50]. Similarly, exergaming interventions have been shown to enhance confidence in other contexts, such as fall-prevention training for older adults, which raised confidence in avoiding falls by 1.05 times [51]. However, only a few studies have directly assessed exergaming’s impact on sports confidence. This trial helps address that gap, showing that tennis-specific exergaming can enhance confidence comparably to on-court training. Notably, there was a study that found exergaming to be less effective than traditional training for psychological outcomes. The trial reported that while exergaming boosted enjoyment and engagement, it did not significantly improve confidence in novice athletes [52]. The difference may reflect variations in exergame type, intervention length, or participant skill level. This study used tennis-specific exergaming directly relevant to participants’ skill goals, while the comparison study [52] used non–sport-specific games.

Overall, the comparable benefits of EBTT+OCTT and OCTT×2 suggest that exergaming can be a valuable addition to sports training, particularly where access to courts or equipment is limited. It may also provide a psychologically supportive environment for beginners or individuals with low self-confidence by reducing the pressures of real-world competition.

#### Effect of Exergaming on Motivation Level

In the EBTT+OCTT group, apart from an increase in amotivation levels, there was a decline in all other motivation categories. Significant decreases were observed only in IM-to-accomplish (by 0.09 times), EM-identified (by 0.22 times), and EM-introjected (by 0.15 times), with medium and small effect sizes, respectively. In the OCTT×2 group, aside from increases in amotivation and IM-to-experience, other motivation levels also declined. Significant decreases were noted in IM-to-know (by 0.28 times), IM-to-accomplish (by 0.24 times), EM-identified (by 0.20 times), and EM–external regulation (by 0.25 times), all with large effect sizes (ηp²=0.12-0.30). These results suggest that both EBTT+OCTT and OCTT×2 may influence IM in complex ways. According to self-determination theory, IM is driven by the inherent satisfaction of performing an activity [53]. The observed decreases in IM components, such as IM-to-accomplish, could suggest that the initial novelty and enjoyment of mastering new skills may diminish over time as tasks become repetitive or as participants reach a plateau in skill development [53]. This aligns with past research showing that continuous, repetitive training can sometimes reduce IM, especially when the perceived challenge or novelty decreases [54]. This pattern is also supported by another study, where motivation level was reduced by 1.08 times in general, and amotivation increased by 3.69 times when the participants were provided with a wearable healthy lifestyle technology (Fitbit by Fitbit Inc) for 8 weeks [55]. These results may indicate that, over time, both intrinsic and EM decline. However, it should be noted that maintaining motivation is essential for sustaining motivation without the need to alter or add new training equipment. Thus, the results from this quasi-experimental study suggest that EBTT+OCTT was able to sustain IM more than OCTT×2.

### The Interrelationship Between Grip Strength, Confidence Level, and Motivation Level Toward an Individual’s Tennis Skills

The interaction between motivation, confidence, and grip strength appears central to enhancing tennis performance. In this study, grip strength showed strong positive correlations with tennis hit score (*r*=0.70), service accuracy (*r*=0.45), and service speed (*r*=0.42). This suggested that stronger grip strength contributed to more powerful and accurate strokes. Similar findings have been reported in previous studies, where grip-strengthening exercises improved forearm speed and service performance [10,20,56]. Stronger grip strength enhances the transfer of force from the lower body and core to the racket, leading to greater ball velocity and control. In this study, participants initially had low grip strength (mean=18.74 kg) and poor Hewitt’s tennis test scores, often falling in the “D” or “F” range. Their early struggles in returning balls accurately highlighted the importance of grip strength for technical execution.

Exergaming, such as traditional training, improved grip strength in this study. The EBTT+OCTT group increased grip strength by 1.5 times, while the OCTT×2 group improved by 1.6 times. Although no significant difference was found between the groups, the repetitive upper-limb motions in exergaming likely contributed to strength gains. Past research supports this, showing that interactive games involving hand and arm actions can build muscular endurance and functional fitness [21,57,58]. The moderate correlation between grip strength and service speed (*r*=0.42) further supports its role in stroke performance. While this study did not establish equivalence between EBTT+OCTT and OCTT×2, the findings suggested that exergaming offered a meaningful, engaging way to strengthen tennis-related muscles.

Confidence also emerged as a critical factor. Posttraining, confidence levels rose significantly in both groups and were positively correlated with tennis hit scores (*r*=0.70) and service speed (*r*=0.33). Confidence helped participants take risks, handle pressure, and execute skills more effectively. Early in training, participants’ low confidence coincided with poor accuracy and hesitancy. By the end of the training, confidence scores improved from 35 of 72 to 61 of 72, accompanied by fourfold increases in hit scores. Previous studies confirm that self-efficacy and confidence are linked to better performance, reduced anxiety, and resilience under stress [48,59-62]. Exergaming may support confidence development by providing progressive challenges and instant feedback, enabling athletes to see measurable progress. This study found confidence increased by 1.6 times in EBTT+OCTT and by 1.8 times in OCTT×2, showing that both methods are effective.

Motivation, particularly IM, also played a key role. Players with high IM were more committed to practice, strengthening both skills and confidence. In this study, IM correlated strongly with hit scores (*r*=0.60, 0.45, and 0.80 for IM-to-know, IM-to-accomplish, and IM-to-experience, respectively), while EM showed weaker links to performance. Although overall motivation decreased slightly over the 12 weeks, the decline was smaller in EBTT+OCTT, suggesting exergaming helped sustain interest. This aligns with previous research showing that task-oriented environments emphasizing skill mastery foster stronger IM than competition-focused settings [63-66]. Exergaming creates such an environment by offering clear goals, instant feedback, and playful competition, all of which help maintain engagement.

Taken together, the results suggest that grip strength, confidence, and motivation interact in a reinforcing cycle. Stronger grip strength enhances stroke performance, which boosts confidence. Increased confidence encourages effort and persistence, feeding back into skill gains. Intrinsic motivation sustains this process by making practice enjoyable and rewarding. Exergaming supports this loop by providing variety, feedback, and achievable challenges, which help maintain both confidence and motivation. While not a full replacement for on-court training, EBTT+OCTT appears to be a valuable complement, especially for novices or those with limited access to facilities. Exergaming requires minimal space, engages players psychologically, and offers cost-effective benefits in both sporting and rehabilitation contexts [49]. Coaches and practitioners could integrate exergaming to enhance early skill acquisition, sustain motivation, and reduce barriers to participation.

### Exergaming Studies in Other Sports and Psychological Domains

The results of this study are consistent with previous exergaming interventions across a range of sports and populations. For example, in a study involving junior athletes practicing basketball, handball, and volleyball, exergaming significantly improved reaction time over 3 months, indicating its utility in sport-specific skill enhancement [67]. Similarly, in a soccer-based exergaming study, players demonstrated improved reaction time and passing accuracy, with larger effects observed in novices compared to experienced athletes [68]. These findings support the results of this quasi-experimental study, whereby novice players may be particularly responsive to exergaming interventions, potentially due to a greater scope for technical and psychological adaptation.

From a psychological perspective, the results align with previous studies showing that exergaming enhances motivation, confidence, and enjoyment. A study found that adolescents who engaged in exergaming reported significantly greater IM and self-efficacy compared to traditional physical education groups [69,70]. Likewise, another study demonstrated that virtual cycling games increased motivation and adherence among older adults [71]. While the effect sizes in this quasi-experimental study were large in terms of motivational outcomes, this may reflect the unique engagement and gamified feedback inherent in exergaming platforms, which are particularly effective in novice or recreational populations. In contrast, elite athletes may show more modest gains due to ceiling effects and previous high-level training.

### Limitations

This study has several limitations that should be acknowledged. First, the findings may only be generalizable to novice players, as the participants recruited had less than 1 year of tennis experience. This may limit the applicability of the results to more experienced or elite players, who may respond differently to exergaming-based interventions. To address this, this quasi-experimental study standardized the training program and ensured participants had similar baseline experience levels. Future studies should explore the effects of exergaming among intermediate or advanced players to assess broader applicability.

Second, the dropout rate was approximately 12% in the OCTT×2 group and 15% in the EBTT+OCTT group. Although this attrition was within acceptable limits for a quasi-experimental design, it could have influenced group comparisons and reduced statistical power. Another key limitation was the group allocation switching that occurred before the start of the experiment, where 7 participants requested to join the group of their preference (OCTT×2 [n=3] and EBTT+OCTT [n=4]). This introduced self-selection bias and could potentially affect the internal validity of the study’s results, especially for outcomes related to motivation. Furthermore, the per-protocol analysis of data reflected the partial allocation of participants’ chosen group, as opposed to randomized groups. This limits the statistical strength and validity of the results reporting motivational outcomes typically associated with randomized designs. As such, findings about motivation in this study should be interpreted with caution, and future studies should consider more rigorous strategies to ensure the robustness of randomization. To mitigate this, the study recruited more participants than the minimum sample size required by power analysis to maintain sufficient statistical validity. Although participants who had requested the group change may have had pre-existing differences in motivation, their baseline levels remained similar compared to those who remained in their allocated groups. In addition, the consistency in results between the modified ITT and per-protocol analyses strengthens the validity of the findings, suggesting that the results are robust to missing data and protocol deviations. However, future studies should incorporate strategies such as participant incentives, participant blinding, or shorter program durations to enhance retention.

Third, the exergaming environment may not fully replicate the physical, cognitive, and environmental demands of real-life tennis. Factors such as court size, surface type, weather conditions, and opponent variability are absent in virtual simulations, potentially limiting the ecological validity of the findings. To address this limitation, EBTT+OCTT participants also received one on-court tennis session per week to complement the virtual training. However, further research should explore advanced platforms (for example, augmented or mixed reality) to better simulate real-world playing conditions.

Fourth, the sex distribution in the study sample was skewed, with approximately 63% female participants. This imbalance may introduce sex-related bias and limit the generalizability of the results across sexes. While the study analyzed group-level performance rather than sex-specific differences, future studies should aim for a more balanced male-to-female ratio or consider conducting sex-stratified analyses.

Fifth, although the study used validated tools to assess motivation, confidence, grip strength, and tennis skills, it relied primarily on quantitative methods. This limit understanding of participant experiences and perspectives regarding the training. Incorporating qualitative methods, such as interviews or focus groups, in future studies could provide richer insight into how and why exergaming may influence motivation and performance. At the same time, the assessor was not blinded to group allocation due to practical constraints inherent in sport-specific field testing, which could introduce potential bias. The tennis skill assessments required direct interaction and real-time scoring, making blinding operationally difficult and largely unfeasible. To minimize bias, a single trained assessor was used to ensure consistent scoring procedures across all participants.

Finally, while no statistically significant differences were found between EBTT+OCTT and OCTT×2 for most outcome measures, this should not be interpreted as evidence of equivalence between the 2 training modalities. The study used standard hypothesis testing and was not designed or powered to formally assess equivalence or noninferiority. Therefore, the finding of nonsignificant *P* values reflects a failure to reject the null hypothesis, not confirmation that the interventions are equally effective. Future studies using equivalence or noninferiority trial designs may be warranted to further evaluate whether EBTT+OCTT can produce outcomes comparable to traditional training.

### Future Directions

Future research should consider several aspects to build upon the findings of this study. First, as mentioned previously, studies should explore the effectiveness of EBTT+OCTT in populations with varying levels of tennis proficiency, including intermediate and elite players, to determine whether skill transfer and psychological benefits persist across experience levels. Second, longitudinal studies with extended follow-up periods are required to assess the sustainability of improvements in technical skills, grip strength, motivation, and confidence beyond the 12-week intervention period. Third, more immersive technologies such as virtual reality or augmented reality could be integrated into exergaming systems to better replicate real-world conditions and improve ecological validity. Fourth, future trials should investigate how exergaming can be personalized based on individual learning styles, physical abilities, or motivational profiles, which may enhance engagement and outcomes. Fifth, incorporating qualitative methods could provide deeper insights into users’ experiences and perceived barriers or facilitators to exergaming adoption. Finally, exergaming could be examined as a rehabilitation tool for individuals with musculoskeletal or neurological impairments, to determine its utility in clinical sports medicine or physiotherapy settings.

### Conclusion

In conclusion, this quasi-experimental study demonstrated that both EBTT+OCTT and OCTT×2 significantly improved tennis technical skills, grip strength, and confidence level among novice players over 12 weeks. EBTT+OCTT also helped sustain participants’ IM level. Although between-group differences were not significant for most outcomes, EBTT+OCTT was more effective in sustaining IM. The observed positive associations between tennis skills, grip strength, confidence, and motivation suggest a correlated process where improvements in one domain support gains in others. Overall, evidence points toward EBTT+OCTT as a feasible and engaging supplementary approach for developing both physical and psychological aspects of tennis performance in novice players. However, given the study’s design and population, conclusions should be interpreted with caution and not generalized beyond novice recreational players. Further controlled studies are needed to confirm the broader applicability and long-term effects of exergaming in sport-specific training.

## Appendix

Multimedia Appendix 1 Results before group reassignment for modified intention-to-treat analysis.

## Abbreviations

EBTT+OCTT: exergame-based tennis training with on-court tennis training
EM: extrinsic motivation
IM: intrinsic motivation
IPAQ: International Physical Activity Questionnaire
ITT: intention-to-treat
MD: mean difference
OCTT: on-court tennis training
SCI: Sports Confidence Inventory
SMT: Sports Motivation Test

## Data Availability

In accordance with the stipulations provided by the ethics governing committee (Universiti Malaya Ethics Committee), all data belongs to Universiti Malaya, and distribution of the data require previous permission from the committee. Study data can be provided upon reasonable request to the corresponding author.
