# Supplementary material for: Effects of Exergaming Tennis on Players’ Tennis Skills and Mental State Compared to Regular Tennis in Adult Players: Quasi-Experimental Study
Source: JMIR Serious Games. 2026 Feb 2;14:e73732. doi: 10.2196/73732 (PMC12910271; doi:10.2196/73732)
Supplement: Multimedia Appendix 1 [file games_v14i1e73732_app1.docx]

**Table S1.** Comparison of grip strength, hit scores, accuracy scores, speed scores, and confidence level within and between groups according to original group allocation.

| Outcome measures | | Pre-test mean (SD) | Post-test mean (SD) | MD^c^ | 95% CI of MD^c^ (Lower, Upper) | Pre-post changes (%) | Within group *P* value | Between groups *P* value |
| --- | --- | --- | --- | --- | --- | --- | --- | --- |
|  | |  |  |  |  |  |  |  |
| **Grip strength** | |  |  |  |  |  |  |  |
|  | EBTT + OCTT^a^ | 21.85 (15.14) | 32.41 (16.01) | 10.56 | 8.78, 12.33 | 48.33 | < .001 | .69 |
|  | OCTT x2^b^ | 16.13 (13.80) | 26.20 (13.88) | 10.07 | 8.29, 11.84 | 62.43 | < .001 |  |
| **Hit scores** | |  |  |  |  |  |  |  |
|  | EBTT + OCTT^a^ | 5.29 (5.51) | 21.85 (7.29) | 16.56 | 14.37, 18.73 | 313.04 | < .001 | .55 |
|  | OCTT x2^b^ | 4.40 (4.64) | 22.00 (9.13) | 17.60 | 14.85, 20.35 | 400.00 | < .001 |  |
| **Accuracy scores** | |  |  |  |  |  |  |  |
|  | EBTT + OCTT^a^ | 4.85 (2.94) | 27.85 (7.86) | 23.04 | 20.20, 25.87 | 475.05 | < .001 | .54 |
|  | OCTT x2^b^ | 4.10 (3.54) | 26.07 (7.90) | 21.97 | 19.77, 24.16 | 535.85 | < .001 |  |
| **Speed scores** | |  |  |  |  |  |  |  |
|  | EBTT + OCTT^a^ | 3.85 (2.72) | 22.48 (6.33) | 18.63 | 16.26, 21.00 | 483.9 | < .001 | .14 |
|  | OCTT x2^b^ | 2.67 (2.29) | 19.10 (5.64) | 16.43 | 14.50, 18.37 | 615.36 | < .001 |  |
| **Confidence level** | |  |  |  |  |  |  |  |
|  | EBTT + OCTT^a^ | 35.63 (8.99) | 61.04 (6.32) | 25.41 | 21.09, 29.73 | 71.32 | < .001 | .70 |
|  | OCTT x2^b^ | 35.53 (7.88) | 59.80 (6.71) | 24.26 | 20.08, 28.46 | 68.28 | < .001 |  |

^a^EBTT + OCTT: Exergame- based tennis training with on-court tennis training (n=27)

^b^OCTT x2: On-court tennis training (n=30)

^c^MD: Mean difference

**Table S2.** Comparison of different motivation levels within and between groups according to original group allocation.

| Outcome measures | | Pre-test mean (SD) | Post-test mean (SD) | MD^c^ | 95% CI of MD^c^ (Lower, Upper) | Pre-post changes (%) | Within group *P* value | Between groups *P* value |
| --- | --- | --- | --- | --- | --- | --- | --- | --- |
|  | |  |  |  |  |  |  |  |
| **IM^d^-to know** | |  |  |  |  |  |  |  |
|  | EBTT + OCTT^a^ | 24.59 (2.75) | 21.96 (4.26) | -2.63 | - 4.73, 0.53 | 10.70 | .02 | .01 |
|  | OCTT x2^b^ | 23.83 (2.65) | 17.77 (3.49) | -6.07 | - 7.84, - 4.30 | 25.47 | < .001 |  |
| **IM^d^-to accomplish** | |  |  |  |  |  |  |  |
|  | EBTT + OCTT^a^ | 23.22 (2.53) | 21.41 (2.50) | -1.81 | - 3.25,  - 0.38 | 7.80 | .02 | < .001 |
|  | OCTT x2^b^ | 24.40 (2.24) | 18.90 (2.51) | -5.50 | - 6.83,  - 4.17 | 22.54 | < .001 |  |
| **IM^d^-to experience stimulation** | |  |  |  |  |  |  |  |
|  | EBTT + OCTT^a^ | 24.59 (2.69) | 23.81 (2.69) | -0.78 | - 2.13, 0.57 | 3.17 | .25 | .19 |
|  | OCTT x2^b^ | 24.13 (2.33) | 24.63 (2.67) | 0.50 | - 0.92, 1.92 | 2.07 | .48 |  |
| **EM^e^–identified** | |  |  |  |  |  |  |  |
|  | EBTT + OCTT^a^ | 24.48 (2.71) | 19.26 (2.52) | -5.22 | - 6.54,  - 3.90 | 21.32 | < .001 | .74 |
|  | OCTT x2^b^ | 24.20 (2.76) | 19.27 (2.68) | -4.93 | - 6.15,  - 3.71 | 20.37 | < .001 |  |
| **EM^e^–introjected** | |  |  |  |  |  |  |  |
|  | EBTT + OCTT^a^ | 16.70 (3.24) | 14.22 (3.00) | -2.48 | - 4.11,  - 0.85 | 14.85 | <.01 | .93 |
|  | OCTT x2^b^ | 17.67 (4.50) | 15.07 (3.18) | -2.60 | - 4.70,  - 0.50 | 14.71 | .02 |  |
| **EM^e^-external regulation** | |  |  |  |  |  |  |  |
|  | EBTT + OCTT^a^ | 24.67 (2.73) | 22.96 (2.78) | -1.70 | - 3.49, 0.08 | 6.89 | .06 | < .001 |
|  | OCTT x2^b^ | 24.23 (2.87) | 18.53 (2.64) | -5.70 | -6.83,  - 4.57 | 23.52 | < .001 |  |
| **Amotivation** | |  |  |  |  |  |  |  |
|  | EBTT + OCTT^a^ | 13.89 (3.20) | 21.44 (2.17) | 7.56 | 5.98, 9.13 | 54.43 | < .001 | .74 |
|  | OCTT x2^b^ | 13.67 (2.95) | 21.57 (2.34) | 7.90 | 6.50, 9.30 | 57.79 | < .001 |  |

^a^EBTT + OCTT: Exergame-based tennis training with on-court tennis training (n=27)

^b^OCTT x2: On-court tennis training (n=30)

^c^MD: Mean difference

^d^IM: Intrinsic motivation

^e^EM: Extrinsic motivation
